# Paths towards Universal Health Coverage: beyond political commitments

**DOI:** 10.7189/jogh.11.16002

**Published:** 2021-11-20

**Authors:** Viroj Tangcharoensathien, Walaiporn Patcharanarumol, Anond Kulthanmanusorn, Ariel Pablos-Mendez

**Affiliations:** 1 International Health Policy Program, Ministry of Public Health, Nonthaburi, Thailand; 2 Division of General Medicine, Columbia University, New York, New York, USA

## Abstract

The rapid economic growth in low and middle-income countries provides the opportunity of translating political commitment into action for achieving Universal Health Coverage. However, this is not straightforward. High donor dependence in low income countries; the lack of fiscal space; the inadequacy of attention to primary health care and under-developed pre-payment systems all pose challenges. Windows of political opportunity open up and ensuring that Universal Health Coverage makes it into the agenda of parties and subsequent holding them accountable by citizens can address political inertia. Not only is more money for health needed, but governments also need to gain more health for money through effective strategic purchasing and addressing the main drivers of inefficiency. Moving Universal Health Coverage from political aspiration to reality requires approaching it as a citizen’s rights and entitlement to health, through full subsidies for the poor and vulnerable.

The changing global health landscape is witnessed by an unprecedented health gain during the era of Millennium Development Goals. The global average life expectancy at birth had surpassed 70 years; though a rich-poor gap remains, it is 63 and 82 years in low- and high-income countries respectively [1]. The global Maternal Mortality Ratio (MMR) fell from 385 deaths per 100 000 live births in 1990, to 216 in 2015, corresponding to a relative decline of 43.9%. In order to achieve Sustainable Development Goal (SDG) targets, countries need to achieve their MMR annual reduction rate of at least 7.5% [2].

While the 5-fold increases in development assistance for health had reached a plateau after the 2008 financial market collapse among donor countries, developing countries have enjoyed a steady economic growth; many of the low-income countries in 2000 reached middle-income status. This provides increased domestic resources for health which can support progressive realization of Universal Health Coverage (UHC) and the achievement of other health-related SDGs [3].

During the last decade, there was a significantly high level of global commitment towards achieving UHC by 2030, through various World Health Assembly resolutions, and by Head of States’ commitments to the United Nations (UN) General Assembly Resolution [4]. At the four-decade review of progress of the 1978 Alma Ata Declaration on Primary Health Care (PHC), the 2018 Astana Declaration reaffirmed countries’ commitments to strengthening PHC as a cornerstone of achieving UHC.

This paper discusses how countries translate the political commitments on UHC from the UN General Assembly High Level Meeting on UHC in September 2019 into reality and proposes suggestions for overcoming challenges in their paths towards UHC.

## THE CHALLENGES

### Inadequate fiscal space for health

In 2017, governments in many low- and middle-income countries were lagging behind in providing adequate health services to the population as committed in the SDG Target 3.8.1 [5] and protecting the population from financial catastrophe [6] and impoverishment due to out-of-pocket payment for health [7] in the SDG Target 3.8.2. Translating these political commitments into reality is achievable though not straightforward.

The inadequacy of PHC hampers access to services needed by all. The lack of pooled prepayment systems limits financial access to the services by the citizens. Most of the low-income countries rely on donor resources which focus on specific diseases rather than health systems strengthening. This is a result of the lack of fiscal space as measured by government revenue or tax as percent of Gross Domestic Product (GDP), as well as inadequate fiscal space for health as measured by public expenditure on health as percent of total public expenditure. All of these challenges are rooted from lack of political and financial commitment by the governments.

**Figure 1:**
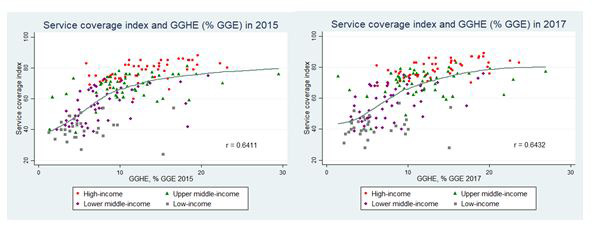
Service coverage index and General Government Health Expenditure as % of General Government Expenditure, 2015 and 2017 [8].

Selected countries in **Table 1** are categorized in two groups, public health expenditure below 5% and above 15% of total public expenditure between 2010 and 2015. In the former group, two countries were seriously affected by the 2013 Ebola outbreaks. Liberia (10 666 cases and 4806 deaths) and Guinea (3804 cases and 2536 deaths) spent only 3% of total public expenditure on health in 2015; their health workforce density was also low, 0.5 physicians, nurses and midwives per 1000 population. Low spending in Bangladesh, Haiti and India are noted. This is in contrast with countries in the second group. There is a positive correlation between government health spending and the service coverage index in 2015 and 2017 (**Figure 1**).

**Table 1:** Public expenditure on health; physicians, nurses and midwives per 1000 population [8]

Country	Public expenditure on health, % total public expenditure						Physicians/1000 pop	Nurses and midwives / 1000 pop		Physicians, nurses and midwives /1000 pop	Service coverage Index	
	**2010**	**2011**	**2012**	**2013**	**2014**	**2015**					**2015**	**2017**
**Group 1 – below 5%:**												
1. Mozambique	1	1	5	3	4	1	0.1 (2013)		0.4 (2013)	0.5	43	46
2. Liberia	4	8	6	5	4	3	0.023 (2010)		0.5 (2010)	0.5	37	39
3. Guinea	2	3	3	3	3	3	0.1 (2016)		0.4 (2016)	0.5	34	37
4. Bangladesh	4	4	4	3	3	3	0.5 (2015)		0.3 (2015)	0.8	46	48
5. Haiti	3	3	3	3	3	3	0.2 (1998)		0.1 (1998)	0.3	47	49
6. India	3	3	4	3	3	3	0.8 (2016)		2.1 (2016)	2.9	52	55
7. Pakistan	3	3	3	3	3	4	1.0 (2015)		0.5 (2015)	1.5	42	45
8. Lao PDR	3	2	2	3	3	4	0.5 (2014)		1.0 (2014)	1.5	49	51
9. Timor-Leste	4	3	4	5	5	4	0.1 (2011)		1.3 (2015)	1.4	49	52
10. Egypt, Arab Rep.	4	5	4	4	4	4	0.8 (2014)		1.4 (2014)	2.2	65	68
**Group 2 – above 15%:**												
1. Madagascar	16	12	10	10	14	16	0.1 (2012)		0.2 (2012)	0.3	24	28
2. Belgium	15	15	15	15	15	16	3.0 (2015)		11.1 (2016)	14.1	83	84
3. Thailand	16	17	17	16	17	17	0.5 (2015)		2.3 (2015)	2.8	75	80
4. Sweden	14	18	18	18	18	18	4.2 (2014)		11.9 (2014)	16.1	85	86
5. High income	17	17	17	18	18	19	3.0 (2013)		8.7 (2013)	11.7	81	82
6. Costa Rica	21	22	21	21	20	19	1.2 (2013)		0.8 (2013)	2.0	76	77
7. Chile	17	18	18	19	19	20	1.0 (2009)		0.1 (2009)	1.1	66	70
8. Iran, Islamic Rep.	12	12	16	16	23	23	1.5 (2014)		1.6 (2014)	3.1	70	72
9. Maldives	14	18	14	22	25	23	3.6 (2015)		8.2 (2015)	11.8	59	62
10. Switzerland	23	23	24	24	24	25	4.2 (2016)		18.2 (2015)	22.4	82	83

Evidence shows that increasing investment in health as share of GDP is not sufficient to reduce the prevalence of catastrophic health spending, but increasing the share of prepayment for health in the GDP, through taxes and mandatory contributions, can reduce catastrophic health expenditure [6] and medical impoverishment [7].

Not only is more money for health needed, but governments also need to gain more health for money through effective strategic purchasing. Greater efficiency can be achieved by applying health technology assessment to prioritize cost-effective interventions; using generic medicines; investing effectively in health promotion and disease prevention; energizing PHC with proper referral mechanisms to hospitals; and minimizing waste in hospitals from inappropriate admissions and lengths of stay, overuse of equipment, investigations and procedures, waste, corruption and fraud [9].

### Inadequate health system capacity

An index of essential services coverage is used for monitoring SDG target 3.8.1. It is a composite index of 16 tracer indicators [5]. In 2015, the index ranged from more than 80 among high-income countries to less than 35 in some conflict-afflicted and low-income countries (**Table 1**).

Not only is the health service coverage index low in some countries, but the lower the index, the higher the rich-poor inequalities [10,11]. Across 52 countries with sufficient data, rich-poor gaps are large. For example, the national level service coverage index in Nigeria and Mozambique was 40 and 42, respectively, while the index for the poorest quintiles was 15 and 17 respectively [5].

An adequate number of committed and competent health workers, particularly in frontline service, is essential in ensuring well-functioning PHC. The low level of the essential service coverage index in **Table 1** results in large part from the low density of the health workforce, as measured by physicians, nurses and midwives per 1000 population, and the high level of health worker absenteeism among many countries in Asia [12] and Africa [13,14]. The density of physicians, nurses and midwives in the first group of countries in **Table 1** is far below the benchmark of 4.45 per 1000 population needed for achieving UHC and health-related SDGs [15]. Government policy needs to scale up health worker training and ensure effective health worker retention in places where they are needed most.

## PATHS TOWARDS UHC

### Overcoming political inertia

A political window of opportunity opens every general election; reformists and civil society organizations should seize this opportunity to encourage political parties to bring UHC into their political manifestos. This advocacy holds governments accountable to their political promises post-election. This was the case in Thailand, where UHC was the political manifesto during the campaign for the January 2001 election and UHC was successfully achieved in 2002 [16]. Success requires citizen ownership of UHC, ensuring fiscal sustainability and protecting it from political interference. Once citizens realize the benefits of UHC, they will protect UHC from political uncertainty and changes of government.

A country should approach UHC as the citizens’ rights and entitlement to health, through full subsidies for the poor and vulnerable. A common dilemma is the source of financing for the informal sector – premium contributions vs general tax. In Thailand, a bold decision was made to finance the Universal Coverage Scheme for citizens outside the formal employment sector through tax. The decision was made not only to honour the political promise during the election campaign, but also for practical reasons. The additional resources were within the government’s fiscal capacity. More importantly, enforcing premium payments by informal workers is not easy and hampers UHC achievement [17]. China is another example where the domestic government budget plays a significant role in achieving UHC.

Though registered migrants can be covered by Social Health Insurance systems in host countries, a difficult political choice remains about whether to extend coverage to a larger number of non-registered migrants and their dependents. Despite their labor and economic contributions to the host countries, protectionism and xenophobia often prevail against such a policy [18]. Universal access to public health interventions such as child immunization and tuberculosis and AIDS treatments among migrants provide health security to the host communities. This justifies the government’s efforts to extend coverage through special migrant health insurance [19].

### Overcoming inefficiency

Strong policy commitment can overcome the ten leading sources of inefficiency [20]. For example, underuse of generic medicines can be resolved by closed-end provider payment such as capitation or diagnostic-related group, as well as incentives for generic substitution which prevent supplier-induced demand. Use of evidence-based clinical guidelines can minimize potential harm and control costs [21]. Use of health technology assessments to decide on the inclusion of cost-effective interventions in the UHC benefit package increases value for money. Health technology assessments are commonly used in European countries [22].

Countries can ensure quality of medicines through post-marketing surveillance to detect substandard and falsified medical products, and though legal actions, if necessary. Use of substandard and falsified antimicrobial drugs can cause antimicrobial resistance and increase mortality and health care costs [23]. Separating the two functions of prescribing and dispensing can prevent potential financial conflicts of interest [24]. The government needs to regulate pharmaceutical market promotional activities which influences physicians’ prescribing behaviors and increase costs for patients or insurance funds.

Evidence shows that provider payment methods such as diagnostic-related groups increase efficiency and transparency and reduce length of stay, though they also financially incentivize premature hospital discharges [25]. This warrants systematic clinical audits of health care providers. Better planning for an optimum hospital capacity can take advantage of economies of scale and prevent low use of certain infrastructure [26].

## CONCLUSION

Enacting UHC legislation or making a political statement at global forums is not enough to bring about access to health services without financial hardship for all citizens. Deliberative efforts to overcome bottlenecks are required in the path towards UHC.

A few factors contribute to the effective implementation of UHC. Extensive geographical coverage of PHC facilitates access to and use of needed health services by all. Prepayment schemes facilitate access to services without financial hardship. Strategic purchasing supports cost containment, efficiency and equity. There is much to learn from various UHC pathfinder countries [27], including both their positive and negative lessons.
